# Malrotation of the Gut: Not Just a Diagnosis of Infancy

**DOI:** 10.7759/cureus.82229

**Published:** 2025-04-14

**Authors:** Sunmeet Singh, Mandy L Maneval, Kiran Sargar, Afif N Kulaylat

**Affiliations:** 1 Family Medicine, Geisinger Lewistown Hospital, Lewistown, USA; 2 Radiology, Geisinger Medical Center, Danville, USA; 3 Pediatric Surgery, Penn State College of Medicine, Hershey, USA

**Keywords:** abdominal pain, gut embryogenesis, gut malrotation, midgut malrotation, volvulus

## Abstract

A congenital malformation of embryonic gastrointestinal (GI) tract development can result in a condition called midgut malrotation, which can cause abdominal obstruction and subsequent acute or chronic GI symptoms. Although historically thought to be a condition of infancy and young children, modern diagnostic techniques have assisted in discovering patients beyond this age group as well. Failure to adequately recognize this condition in adolescents and adults has resulted in delayed or missed diagnoses, patient harm, and life-threatening complications of this condition, such as volvulus. We present the case of an adolescent experiencing three weeks of abdominal pain, along with other GI symptoms, caused by midgut malrotation.

## Introduction

Midgut malrotation is a defect in the normal embryonic rotation of the gastrointestinal (GI) tract, leading to abdominal obstruction. This may cause acute or chronic GI symptoms ranging from simple abdominal pain, distension, nausea, vomiting, and diarrhea to more life-threatening complications, such as volvulus. It has previously been thought that approximately 90% of malrotation cases are diagnosed in the first year of life and that 80% of those within the first month; however, some reports now suggest that only around 50% are diagnosed within the first year [1]. Furthermore, autopsy reports suggest that this condition may be present in up to 1% of the population. Thus, it is important to remember that this diagnosis may also initially present later in life and should be considered in adolescents and adults with acute, chronic, or intermittent GI symptoms [2]. Failure to adequately recognize this condition may result in misdiagnosis, patient harm, and even life-threatening conditions, such as volvulus.

Intestinal rotational anomalies occur when the normal rotation of the embryonic gut is arrested. GI organogenesis begins at three to eight weeks in the human embryo. The midgut spans the length of the distal duodenum to the proximal two-thirds of the transverse colon and is perfused by the superior mesenteric artery (SMA). In a healthy individual, the gut grows and penetrates through the yolk sac at six weeks. Between four and eight weeks, the small bowel rotates in a counterclockwise direction around the SMA axis for a total of 270 degrees, which is completed at 12 weeks. An early arrest in this normal rotation leads to malrotation [2,3]. In children, it may also be associated with other conditions, such as trisomy 21, along with cardiac and genitourinary anomalies. It is estimated to be present in one in 6,000 live births with symptomatic consequences; however, it is likely present far more often than detected, with the assumption that most individuals remain asymptomatic throughout their lifetime [2,4].

## Case presentation

A 16-year-old previously healthy male presented to his primary care physician complaining of three weeks of epigastric and periumbilical abdominal pain, accompanied by daily non-bloody, non-bilious emesis, non-bloody diarrhea, belching, flatulence, and a reduced appetite. The symptoms were severe enough to leave him bedbound for three days, followed by a week of improvement and then recurrence. He denied experiencing fevers, chills, chest pain, shortness of breath, upper respiratory infection (URI)-like symptoms, or exposure to new foods or animals. At presentation, his vital signs were normal, and the abdominal exam was significant for periumbilical tenderness with mild rebound pain. Laboratory evaluation included complete blood count (CBC), comprehensive metabolic panel (CMP), erythrocyte sedimentation rate (ESR), and urinalysis, which were normal, except for mild eosinophilia, which was likely an incidental finding and not pertinent to the case presentation (Table 1).

**Table 1:** Patient's laboratory values

Parameters	Patient Values	Reference Range
Sodium	140	135-145 mg/dL
Potassium	4.3	3.5-5.0 mEq/L
Chloride	103	95-107 mEq/L
Co-2	25	24-31 mEq/L
Alkaline Phosphatase	103	36-210 IU/L
Alanine aminotransferase (ALT)	11	10-40 IU/L
Aspartate aminotransferase (AST)	15	3.0-42 IU/L
Total Bilirubin	0.5	0.1-1.3 mg/dL
Calcium	10.1	8.5-10.6 mg/dL
Total Protein	7.4	5.8-8.0 g/dL
Albumin	5	3.0-5.2 g/dL
Glucose	85	70-110 mg/dL
Blood Urea Nitrogen	14	6-25 mg/dL
Creatinine	0.7	0.7-1.2 mg/dL
Glomerular Filtration Rate	160	>60 mL/min/1.73 m^2^
White Blood Cell Count	5.7	3.1-9.2 (x10^3^/mm^3^)
Hemoglobin	15.3	12.5-17.5 g/dL
Hematocrit	45.5	37.5-52.5 %
Platelets	187	140-350 (x10^3^/mm^3^)
% neutrophils	42.1	40-75 %
%lymphocytes	42.2	17-45 %
%monocytes	8.7	1.0-11 %
%eosinophils	6.3	0-6 %
Erythrocyte Sedimentation Rate	1	0-15 mm/hr

**Figure 1:**
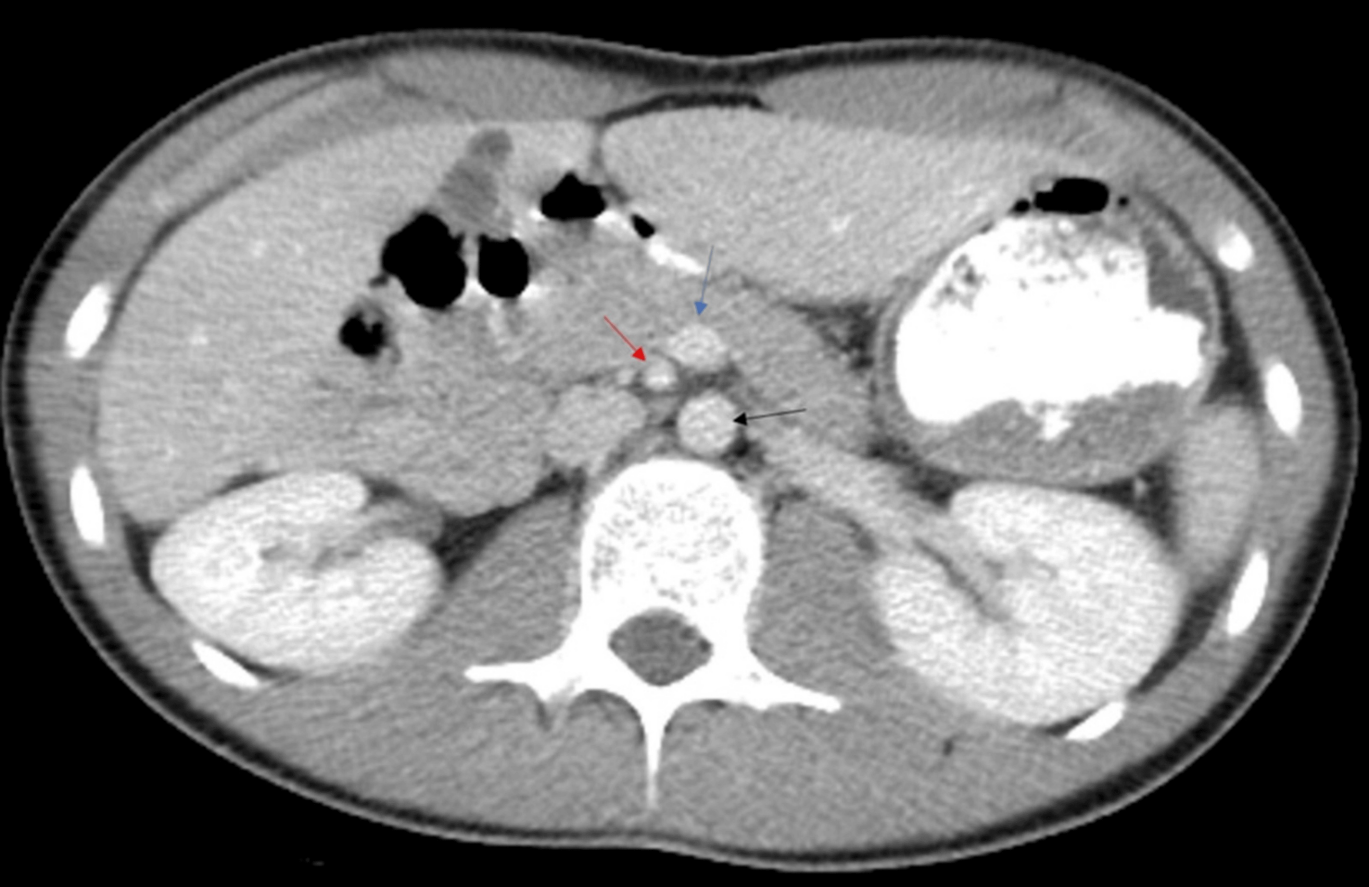
Axial contrast-enhanced CT image of the upper abdomen This image reveals a reversal of the normal relationship of the superior mesenteric artery (SMA, red arrow) and superior mesenteric vein (SMV, blue arrow), with SMV lying on the left side of the SMA. Note that the duodenum is not seen crossing between the aorta (black arrow) and the SMA (red arrow).

**Figure 2:**
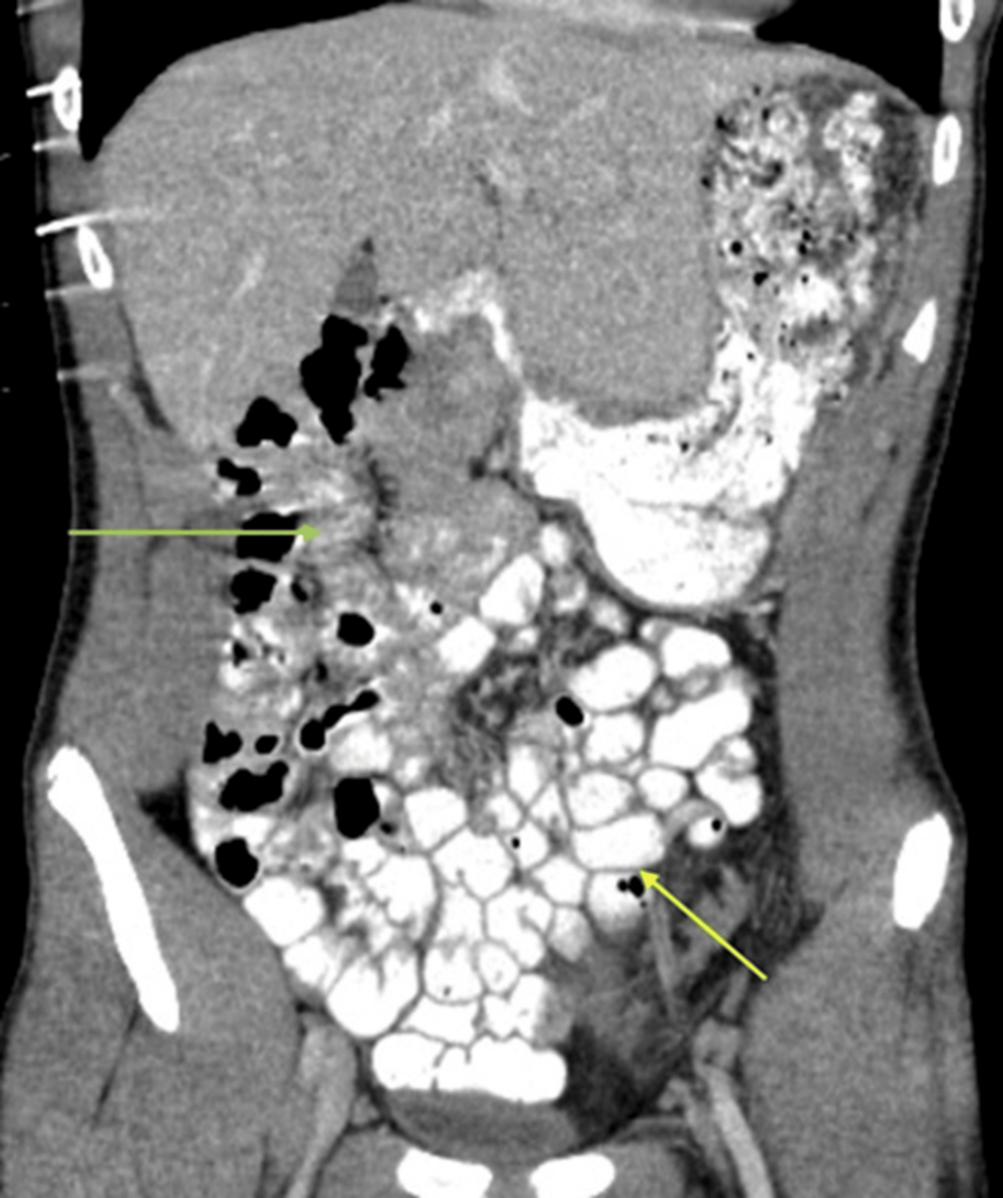
Coronal CT image of the abdomen This image shows the jejunal loops (green arrow) in the right upper abdomen and ileal loops (yellow arrow) in the left lower and mid abdomen.

A GI pathogen panel was also negative for common infectious pathogens. CT of the abdomen and pelvis with contrast was ordered for suspicion of acute appendicitis, or other abdominal pathology, and revealed midgut malrotation without volvulus. Specifically, CT showed failure of crossing of the third portion of the duodenum across the junction of the SMA and aorta. The proximal small bowel loops were seen on the right side of the abdomen, with large bowel loops seen on the left side. The normal relationship of the SMA and superior mesenteric vein (SMV) was reversed (Figures 1-2).

**Figure 3:**
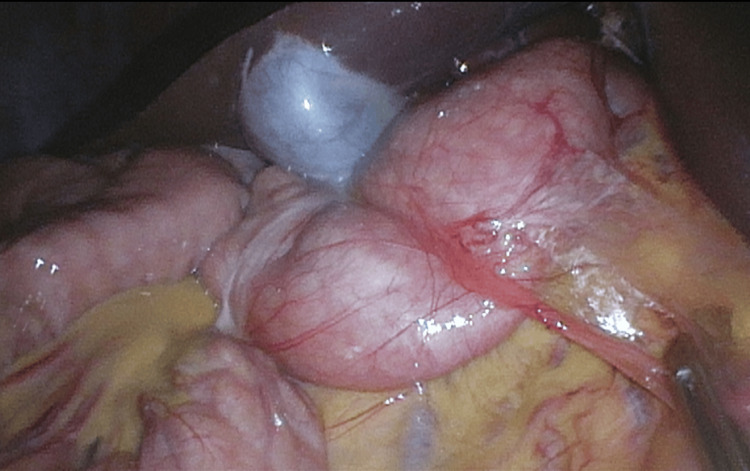
Intraoperative abdominal photo This photo shows Ladd’s bands folding a redundant duodenum medially and then progressing to the jejunal loops that are inferior to the gallbladder, corresponding to the green arrow seen in Figure 2.

The patient was referred to pediatric surgery and underwent a laparoscopic Ladd procedure, which resolved his abdominal pain and other symptoms (Figure 3).

## Discussion

The differential diagnosis of acute and chronic GI symptoms is extensive and includes infectious and inflammatory etiologies, irritable bowel disease, constipation, bowel obstruction, and even malignancy. The diagnostic and therapeutic approach often focuses on the age of the patient and the duration of symptoms, along with associated physical examination findings. The acuity of our patient's symptoms and the suspicion of acute appendicitis led to a CT scan and a subsequent diagnosis of midgut malrotation. There were no associated lab findings to suggest such a serious etiology, and this diagnosis might have been missed in the absence of imaging. Though our patient was promptly diagnosed, the common belief that midgut malrotation likely only presents in infancy may also contribute to a delay in diagnosis in some patients.

The presentation of this condition varies based on the patient's age. In children under one year of age, malrotation commonly presents with emesis, usually bilious. In adolescents and adults, it is often associated with vague, nonspecific, chronic symptoms, such as abdominal pain, cramps, nausea, and vomiting. Less commonly, it may present with constipation, intermittent diarrhea, hematochezia, peptic ulcer disease, malabsorption, early satiety, and weight loss [4]. The diagnosis is often made more promptly in children than adults. The majority of adults experience symptoms for six months or more and are more likely to be misdiagnosed [2]. Although the chronic symptoms may be related to intermittent bowel obstruction from the congenital adhesions (Ladd’s bands), malrotation may also present as a life-threatening complication of volvulus. This occurs when the bowel twists around the SMA in a clockwise rotation, causing acute bowel obstruction and resulting in infarction. Unless treated with emergent corrective surgery, volvulus poses a significant risk of morbidity and mortality. There are no diagnostic modalities that can accurately assess the risk of midgut volvulus in an intestinal rotational anomaly [2].

The preferred method for diagnosing midgut malrotation in pediatric patients is a fluoroscopic upper GI (UGI) series, which has a sensitivity and specificity of 91% and 94%, respectively [5]. The diagnostic criteria include the failure of crossing of the duodenojejunal (D-J) junction to the left of the vertebral pedicle and inferior to the level of the duodenal bulb, with jejunal loops located in the right upper quadrant in the frontal views. Abdominal ultrasound may provide additional supportive or diagnostic information. For this modality, diagnostic criteria in children include an inverted relationship of the mesenteric vessels, duodenal dilation, and the “whirlpool sign,” which is the swirling appearance of bowel and the SMV twisted around the SMA axis. Ultrasound has a sensitivity and specificity of 88% and 96%, respectively, for diagnosing intestinal obstruction and may have a more reliable role in the diagnosis of volvulus [5,6]. The important advantage of ultrasonography in the pediatric population is avoiding ionizing radiation. In adults, however, CT of the abdomen is the more common modality for diagnosing malrotation.

The mainstay of treatment for midgut malrotation, with or without volvulus, is surgery. The most common approach is the Ladd procedure, which involves the counterclockwise detorsion of the midgut volvulus (if present), division of the Ladd's bands, and widening of the mesenteric base to prevent future occurrence or recurrence of volvulus [7]. This may also be accompanied by a prophylactic appendectomy to prevent diagnostic confusion in the future. When performed laparoscopically, this procedure has a low mortality and can be safely performed in patients of all ages [2]. In patients with low surgical risk, surgical correction of malrotation is recommended, even in patients with minor symptoms or an incidental discovery, to prevent life-threatening complications, such as volvulus [4,7]. Complications of this procedure include recurrent volvulus, small bowel obstruction, wound infections, and deep vein thromboses, along with aspiration pneumonia secondary to NG tube placement for small bowel decompression [6]. Adult patients have a higher rate of major complications than children, likely due to the presence of more comorbidities or difficulty tolerating unanticipated or long surgery. The benefit of a Ladd procedure should be weighed on a case-by-case basis [2,8].

## Conclusions

The differential diagnosis for acute and chronic abdominal symptoms is extensive. Previously, malrotation of the gut was associated mostly with infancy; however, as our case demonstrates, malrotation of the gut should be on the differential when adolescents or adults present with either acute or chronic GI symptoms of unknown etiology. Limited awareness and suspicion of malrotation, as a potential etiology for common abdominal complaints, may lead to delays in diagnosis and treatment, with volvulus being the most life-threatening possibility. The diagnostic imaging modality of choice depends on the age of the patient, and surgery remains the mainstay of treatment for most cases of malrotation. Once detected, surgery is often curative.
